# Prevention of Biofilm Formation and Removal of Existing Biofilms by Extracellular DNases of *Campylobacter jejuni*

**DOI:** 10.1371/journal.pone.0121680

**Published:** 2015-03-24

**Authors:** Helen L. Brown, Mark Reuter, Kate Hanman, Roy P. Betts, Arnoud H. M. van Vliet

**Affiliations:** 1 Institute of Food Research, Norwich Research Park, Colney Lane, Norwich, NR4 7UA, United Kingdom; 2 Campden BRI, Station Road, Chipping Campden, Gloucestershire, GL55 6LD, United Kingdom; Robert Koch-Institute, GERMANY

## Abstract

The fastidious nature of the foodborne bacterial pathogen *Campylobacter jejuni* contrasts with its ability to survive in the food chain. The formation of biofilms, or the integration into existing biofilms by *C*. *jejuni*, is thought to contribute to food chain survival. As extracellular DNA (eDNA) has previously been proposed to play a role in *C*. *jejuni* biofilms, we have investigated the role of extracellular DNases (eDNases) produced by *C*. *jejuni* in biofilm formation. A search of 2791 *C*. *jejuni* genomes highlighted that almost half of *C*. *jejuni* genomes contains at least one eDNase gene, but only a minority of isolates contains two or three of these eDNase genes, such as *C*. *jejuni* strain RM1221 which contains the *cje0256*, *cje0566 *and *cje1441 *eDNase genes. Strain RM1221 did not form biofilms, whereas the eDNase-negative strains NCTC 11168 and 81116 did. Incubation of pre-formed biofilms of NCTC 11168 with live *C*. *jejuni* RM1221 or with spent medium from a RM1221 culture resulted in removal of the biofilm. Inactivation of the *cje1441* eDNase gene in strain RM1221 restored biofilm formation, and made the mutant unable to degrade biofilms of strain NCTC 11168. Finally, *C*. *jejuni* strain RM1221 was able to degrade genomic DNA from *C*. *jejuni* NCTC 11168, 81116 and RM1221, whereas strain NCTC 11168 and the RM1221 *cje1441* mutant were unable to do so. This was mirrored by an absence of eDNA in overnight cultures of *C*. *jejuni* RM1221. This suggests that the activity of eDNases in *C*. *jejuni* affects biofilm formation and is not conducive to a biofilm lifestyle. These eDNases do however have a potential role in controlling biofilm formation by *C*. *jejuni* strains in food chain relevant environments.

## Introduction

A biofilm is defined as a mono-species or multi-species population of bacterial cells, which is attached to a surface and surrounded by an extracellular polymeric substance (EPS) [1]. The matrix composition is highly variable, and is dependent on the microbial species populating the biofilm, but generally contains nucleic acids, proteins and polysaccharides [2]. The EPS is an essential component of the bacterial biofilm, and can account for up to 90% of its dry mass depending on microbial species and specific isolates [3]. One frequently found component of EPS is extracellular DNA (eDNA), which plays an important structural role in biofilms, and the addition of exogenous DNase enzymes such as DNase I can disrupt biofilm formation and maturation [4, 5, 6, 7]. Some bacteria are able to secrete their own DNase enzymes into the extracellular environment (hereafter these enzymes are referred to as eDNase). Depending on the bacterial species, these eDNase proteins have diverse functions, such as immune evasion [8, 9], biofilm modification [10, 11], scavenging of carbon and phosphate sources [12, 13], efficient bacterial predation [14], and inhibition of natural transformation [15, 16].

*Campylobacter jejuni* is a leading cause of bacterial foodborne poisoning, in the UK alone there are up 80,000 confirmed cases annually, however underreporting of cases is known to be a problem and the actual figure is estimated to be up to nine times higher than the reported numbers [17]. Infections can be severe but are typically self-limiting. An important impact of *Campylobacter* infection in developed countries is economic, although infection may also lead to significant post-infectious consequences such as Guillain–Barré syndrome [18]. The high incidence of *Campylobacter* infection is surprising in view of the fastidious nature of *C*. *jejuni*, which requires microaerobic and capnophilic conditions, and a narrow temperature range of 37°C to 42°C to grow optimally [19]. *C*. *jejuni* is able to persist for relatively long periods on food and in the environment, and biofilms, or surface attachment, are thought to contribute to persistence [20, 21, 22, 23].

*C*. *jejuni* has previously been shown to be capable of forming biofilms and can also colonise pre-existing biofilms [24, 25], although the levels of biofilm formation varies between isolates [20, 23, 26]. *C*. *jejuni* is a genetically diverse species [27], and insertion elements and prophages are important elements contributing to this diversity. Four of these insertion elements (CJIE1 to CJIE4) were first described in the chicken isolate RM1221 [28, 29], and three of these (CJIE1, CJIE2 and CJIE4) contain genes encoding DNase proteins (*cje0256* (*dns*), *cje0556* and *cje1441* respectively). The encoded proteins are predicted to be extracellular due to the presence of signal peptide cleavage site [15], and their expression prevents natural competence of strain RM1221 [15, 16].

The contribution of biofilms to *C*. *jejuni* transmission through the food chain is becoming apparent [19, 25], and several genetic factors contributing to biofilm formation have been identified in *C*. *jejuni* [30, 31, 32]. There is however still relatively little known about the structure and composition of the *C*. *jejuni* biofilm EPS. Since eDNA is important in *C*. *jejuni* biofilm formation and maturation [33], and DNase I was able to reduce the levels of biofilm of a *C*. *jejuni* 81–176 Δ*cprS* mutant [30], we speculated that the eDNases may also modulate biofilm formation. In this study we have investigated the impact eDNase enzyme activity may have on *C*. *jejuni* biofilm formation. We have investigated the distribution of eDNase genes in a large collection of *C*. *jejuni* genome sequences, and show that eDNase genes are found in almost half of *C*. *jejuni* isolates. We present phenotypic and genetic data that demonstrate that eDNase activity in *C*. *jejuni* RM1221 results in degradation of existing biofilms, and can also prevent biofilm formation by *C*. *jejuni* isolates lacking eDNase genes.

## Materials and Methods

### *C*. *jejuni* strains and growth conditions

A list of *C*. *jejuni* strains and primers used in this study is given in Table 1. *C*. *jejuni* strains were routinely cultured in a MACS-MG-1000 controlled atmosphere cabinet (Don Whitley Scientific) under microaerobic conditions (85% N_2_, 5% O_2_, 10% CO_2_) at 37°C. For growth on plates, strains were either grown on Brucella agar or BAB with Skirrow supplements (10 μg/ml vancomycin, 5 μg/ml trimethoprim, 2.5 IU polymyxin-B). Broth culture was carried out in Brucella broth (Becton & Dickinson). An Innova 4230 (New Brunswick Scientific) incubator was used for aerobic culture at 37°C.

**Table 1:** List of bacterial strains, plasmids and primers used in this study.

Name	Specification	Source
**Bacterial Strains (*C*. *jejuni* unless indicated otherwise)**		
NCTC 11168	Wild-type	[34]
NCTC 11168 Δ*flaAB*	Δ*cj1338*, *Δcj1339c*)::kan^R^	[35]
NCTC 11168 GFP	*cj0046*::Promoter_porA_-GFP^+^::Cat^R^	This study
81116	Wild-type	[36]
RM1221	Wild-type	[28]
RM1221 Δ*1441*	Δ*cje1441*::Cat^R^	This study
*E*. *coli* Top 10	General cloning strain	Invitrogen
*E*. *coli* M147	Non-methylating *E*. *coli* strain (*dam dcm gal ara lac thr leu thi tonA tsx rpsL*)	[37]
**Plasmids**		
pNEB193	General subcloning vector. High copy number Amp^R^, in frame *lacZ*α-complementing vector	New England Biosciences
pCporAGFP^+^	*cj0046*::Promoter_porA_-GFP^+^::Cat^R^ in plasmid pC46 [38]	Duncan Gaskin (IFR)
pET28a	T7 promoter expression plasmid. Used in this study for DNase assays.	Novagen
**Primers**		
1441KO_FDEcoRI	5’-GCATTGAAAGAATTCTATGAGTTAAAAAAGG-3’	This study
1441KO_RVPstI	5’-GCTTTTTAACGCTGCAGTTGATAGGTTGT-3’	This study
1441KO_2_fwd	5’-ATAGGATCCGTTACCAAGTGCCTAATCAC-3’	This study
1441KO_2_rev	5’-ATAGGATCCGGTTTGTATTGTGTATAATC-3’	This study
1441_fwd_schk2	5’-GGAAAATTATTATGAATTAG-3’	This study
1441_rev_schk2	5’-GCCAATAGCAAAAAATGAAC-3’	This study
GFP fwdreadin	5’-GGAGAAGAACTTTTCACTGGAGTTG-3’	This study
GFP revreadin	5’-GCAGTTACAAACTCAAGAAGGACC-3’	This study
cat_rev_readin	5’-GGACACGAAAAGAGTATTTCGACC-3’	This study
cat_fwd_readin	5’-GCATGATGCACTTGAATCGATAAGG-3’	This study

### *Campylobacter* growth for biofilm assay

*C*. *jejuni* culture for biofilm formation was carried out as described previously [35]. Briefly, *C*. *jejuni* from Skirrow plates were used to inoculate Brucella broth then grown overnight as a shaking culture (37°C, microaerobic conditions). Following overnight growth, cell cultures were adjusted to an A_600_ of 0.05 in Brucella medium. To allow biofilm formation, 1 ml of this solution was added to a sterile borosilicate glass test tube (Corning). Tubes were incubated at 37°C in either microaerobic or atmospheric air conditions for 48 hours before staining.

For biofilm formation on glass slides, 20 ml of *C*. *jejuni* culture of A_600_ = 0.05 was added to a 50 ml tube (Corning) containing a sterile twin frost borosilicate glass microscope slide (VWR) and incubated statically at 37°C for 48 hours. Following incubation the slide was gently washed in sterile water and fixed by incubation in 4% formalin for 15 minutes before drying. Slides were stored at 4°C in the dark until use.

Biofilm degradation by strain RM1221 was performed by allowing biofilms to form for 24 h before adding a second (1 ml) volume of either fresh Brucella medium or diluted cell suspension. Biofilm cultures were then incubated for a further 24 h before viability assessment and crystal violet staining. Where spent media was used in the secondary incubation, instead of cell suspensions, the spent medium was prepared from overnight cultures of *C*. *jejuni*. The cells were subsequently pelleted by centrifugation, and the supernatant filter-sterilised using a 0.2 μm polyethersulfone filter, and frozen at -20°C until required.

### Crystal violet staining

Cell suspensions were removed, and the tubes were washed with water and dried at 60°C for 30 min, followed by addition of 1 ml of 1% w/v crystal violet solution. Tubes were further incubated on a rocker at room temperature for 30 min. After incubation, the non-bound dye was removed from the tubes by thorough washing in water followed by drying at 37°C. Bound crystal violet was dissolved by adding 20% acetone/80% ethanol and incubating on a rocking platform for 15 min at room temperature. The resulting dissolved dye was measured at a wavelength of 590 nm using a Biomate 5 spectrophotometer (Thermo Scientific) [39].

### Assessment of cell viability by culture

To determine the number of viable cells, the planktonic fraction was eight-fold serially diluted in PBS and 5 μl of each dilution spotted on Brucella agar plates. After two days of growth in microaerobic conditions, the dilution resulting in two or more colonies was recorded. Cell viability in biofilm assays was assessed upon initial addition of cultures into static culture and following static incubation, prior to crystal violet staining.

### Creation of a *C*. *jejuni* strain expressing green fluorescent protein

To generate a strain of *C*. *jejuni* that constitutively expressed GFP protein, strain NCTC 11168 was transformed with plasmid pCporAGFP^+^ using standard protocols [40]. Plasmid pCporAGFP^+^ contains the *gfp* gene from pWM1007 [41] under control of the *C*. *jejuni porA* promoter and a chloramphenicol resistance cassette, flanked by the 5' and 3' sequences of the *cj0046* pseudogene [38]. Replacement of the *cj0046* pseudogene with the GFP gene and chloramphenicol cassette was confirmed using the primers GFP fwdreadin, GFP revreadin, cat fwd readin and cat rev readin. Fluorescence was assessed by microscopy using a Zeiss 200M fluorescent and light microscope with Axiovision software.

### DAPI staining of NCTC 11168 GFP biofilms

Biofilms previously grown on glass slides for 48 h were allowed to equilibrate to room temperature in dark, aerobic conditions, before staining with 4',6-Diamidino-2-Phenylindole Dihydrochloride (DAPI) using manufacturers guidelines (Invitrogen). Prior to addition of a coverslip, Slowfade Gold antifade reagent (Invitrogen) was added to the slide as recommended by the manufacturer. Slides were imaged using a Zeiss 200M fluorescent and light microscope with Axiovision software.

### Visualisation of extracellular DNA from biofilms

Following static incubation to allow biofilm formation in microaerobic conditions, the supernatant was removed and the tubes were rinsed once with sterile PBS to remove loosely attached bacterial populations. Adhered cells were recovered from the surface of six borosilicate tubes and pooled: 1 ml of sterile PBS was added to the first tube and the adhered cells were gently resuspended using a sterile cotton wool swab. This suspension was removed and used to resuspend adhered cells from a second tube. This was repeated for all six tubes. The A_600_ of the biofilm suspension was recorded, and the cells were diluted in sterile PBS to an A_600_ of 0.3. A 20 μl aliquot of cells was mixed with 4 μl 6× gel loading buffer and loaded on a 0.9% agarose gel. A 1 kb ladder (NEB) was used for size comparison. Following 45 minutes electrophoresis in 0.5% TBE buffer at 100 V, the gel was stained with ethidium bromide, and DNA was visualised using a GelVue UV light and documented using a U:Genius gel documentation system (Syngene).

### Creation of the *C*. *jejuni* RM1221 Δ*cje1441* mutant

A *C*. *jejuni* RM1221 *cje1441* mutant (hereafter referred to as Δ*1441*) was created by insertional inactivation of the *cje1441* gene with a chloramphenicol resistance cassette. The *cje1441* gene and flanking regions were PCR amplified using the primers 1441KO_RVPstI and 1441KO_FDEcoRI and cloned into the pNEB193 plasmid (NEB). Subsequently the *cje1441* was replaced with the *cat* cassette from pAV35 [40] by inverse PCR using primers 1441KO_2_fwd and 1441KO_2_rev. As strain RM1221 is non-transformable due to eDNase expression [16], *in vitro* methylation of the suicide plasmid was used to increase transformation efficiency [42]. Prior to electroporation, RM1221 cells were incubated on ice in 15% (v/v) glycerol, 272 mM sucrose, containing 10 mM EDTA for 1 hour. Following incubation the cells were washed with 15% glycerol, 272 mM sucrose to remove the EDTA and transformed using standard procedures [40].

### Assessment of swarming and autoagglutination

Motility of *C*. *jejuni* was assessed on 0.4% agar plates, as described previously [38]. Briefly, *C*. *jejuni* overnight culture (5 μl) was spotted onto Brucella medium supplemented with 0.4% agar and 0.05% TTC (2,3,5 triphenyltetrazolium chloride) before incubation at 37°C in microaerobic conditions for 48 h. The size of the halos were measured and compared to show relative motility between strains and mutants tested. Autoagglutination was measured as described previously [43] by monitoring the decrease in A_600_ over a 24 h period following incubation in a cuvette at room temperature in aerobic conditions.

### Degradation of extracellular DNA by *C*. *jejuni* RM1221

Degradation of exogenous DNA was investigated using two separate experimental approaches: assessment of a) eDNA degradation by *C*. *jejuni* RM1221 during growth and b) the ability of *C*. *jejuni* RM1221 to degrade purified DNA over a fixed time period. To assess eDNase activity in the supernatant of growing cultures, overnight cultures of *C*. *jejuni* were pelleted and an aliquot of the supernatant was removed for DNase activity assessment. DNase I (Fermentas) and RNase (QIAgen) treatments were carried out following manufacturers guidelines and incubated at 37°C in a water bath for one hour.

Degradation of purified DNA by *C*. *jejuni* strains NCTC 11168, RM1221 and the RM1221 Δ*1441* mutant was also assessed over a fixed time period. *C*. *jejuni* RM1221 cells were allowed to form a lawn on Skirrow plates. The cells were removed from the plate and suspended in 2 ml Brucella medium before pelleting and washing twice in sterile PBS. Following washing, the cell concentration was measured and the culture diluted to an A_600_ of 0.5 in sterile PBS. To digest purified genomic DNA, 50 μl of cell suspension was added to approximately 2 μg of genomic *C*. *jejuni* NCTC 11168 DNA, and incubated at 37°C in a water bath for up to three hours. At 30 min intervals, an aliquot was taken, the cells pelleted and the supernatant removed and frozen at -20°C until analysis. For degradation of plasmid DNA, plasmid pET28a was purified from either *E*. *coli* strain Top10 (*dam* ^+^ *dcm* ^+^) or M147 (*dam* ^-^ *dcm* ^-^) using a commercial miniprep kit (QIAGEN). To generate linear DNA, a 999 bp fragment was amplified from *C*. *jejuni* NCTC11168 genomic DNA with primers cj1388comp_Fwd (5'-GGAGAATTCATGTCAAACTATCCAAAG-3') and pCASO51gDNARevScreen (5’-CCTACAGCTATAATGATAGGCAAGG-3’) using HotStarTaq (QIAGEN). DNA concentration was determined using a Nanodrop 2000 spectrophotometer (Thermo Scientific). Assays contained 70 ng DNA substrate and 1 μl cell suspension in a total volume of 10 μl. EDTA was added at a final concentration of 50 mM. DNase (Fermentas) was added at a final concentration of 1 U and reactions were supplemented with 1x DNase buffer (2.5 mM MgCl_2_). Prior to electrophoresis, samples were mixed with 6x gel loading buffer and loaded onto a 0.9% agarose gel. A 1 kb ladder (NEB) was used for size comparison. Following 45 minutes electrophoresis in 0.5% TBE buffer at 100 V, the gel was stained with ethidium bromide, and DNA was visualised using a GelVue UV light and documented using a U:Genius gel documentation system (Syngene).

### DNase I treatment of *C*. *jejuni* RM1221 Δ*1441* biofilms

Biofilms were grown for 48 h in glass tubes. A volume of 4 μl DNase I, to give a final concentration of 4 U/ml (Fermentas) and 4 μl DNase I buffer (Fermentas) was added to test tubes at the start of the incubation. Following treatment, biofilms were either re-incubated for the remaining 48 h incubation, washed and crystal violet stained or washed and a new volume of cell culture or Brucella medium added to the tube.

### Identification of DNase-encoding genes in *C*. *jejuni* genome sequences

A total of 16 complete and 2781 draft genome sequences of *C*. *jejuni* were obtained from public collections such as pubMLST (http://pubmlst.org/campylobacter/) [44] (N = 2687), and the NCBI (http://www.ncbi.nlm.nih.gov/genome/browse/), and the Virginia Tech University PATRIC website (http://patricbrc.vbi.vt.edu/portal/portal/patric/Home) (N = 104) [45], and are listed with accession numbers and assembly status (S1 Table). Genomes were searched using MIST [46] and the BLAST+ (v2.28) suite with each individual gene of *C*. *jejuni* RM1221 CJIE1 (*cje0213*-*cje0273*), CJIE1 (*cje0544*-*cje0601*) and CJIE4 (*cje1418*-*cje1474*) [28]. Genes were considered to be present if matching ≥ 90% with the query sequence. Genomes were scored as positive for *cje0256*, *cje0566* or *cj1441* if a positive score for the respective gene was matched with ≥ 60% of the respective CJIE1, CJIE2 or CJIE4 genes being present. The MLST-clonal complex designation was determined for all genomes using MIST, with the definition file provided by the Campylobacter pubMLST website.

### Statistical analysis

Statistical analysis was carried out using GraphPad Prism. At least three biological replicates (each with three technical replicates) were used to calculate median and interquartile range. Significance was measured using Mann-Whitney tests.

## Results

### DNase-encoding genes are differentially distributed in *C*. *jejuni* strains

**Fig 1:**
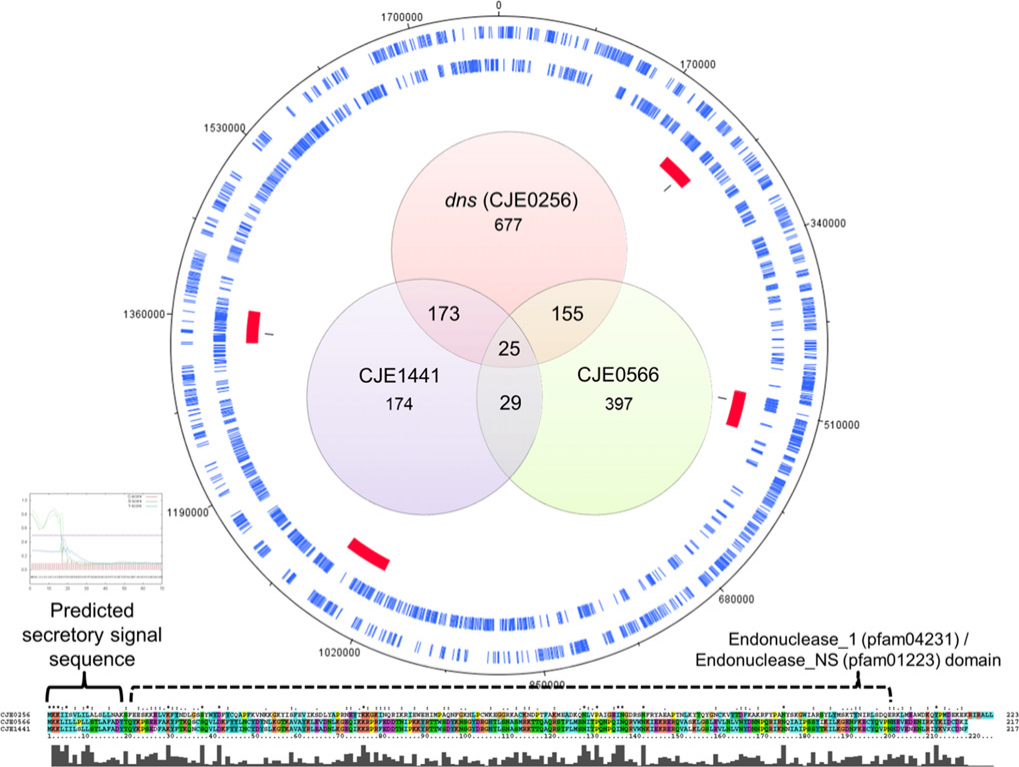
Distribution of eDNase genes in *C*. *jejuni* genome sequences. The Venn diagram shows the distribution of the eDNase genes *dns* (*cje0256*), *cje0566* and *cje1441* in the genome sequences of 1630 eDNase gene-positive *C*. *jejuni* strains (Table 2). Most genomes (1248 of 1630) only have a single eDNase gene, 357 genomes have two eDNase genes, while only 25 genomes, including *C*. *jejuni* RM1221, contain all three eDNase genes. The Venn diagram is encircled by the RM1221 chromosome showing open reading frames (blue), CJIE1–4 insertion elements (red), and the position of the three eDNase genes (black). Finally, the bottom part shows an amino acid sequence alignment of the Dns, CJE0566 and CJE1441 proteins, with the signal sequence and Pfam domains indicated. Signal sequences were predicted using PSORTb version 3.0.2.

We investigated the distribution of eDNase genes in a collection of 2791 publicly available *C*. *jejuni* genome sequences, using the *C*. *jejuni* RM1221 CJIE1, CJIE2 and CJIE4 elements including the eDNase genes *dns* (*cje0256*), *cje0566* and *cje1441* as query sequences (Table 2). Of these genomes, 42% lacked any of the three eDNase genes. Orthologs of the *dns* gene were detected in 37% of genomes, whereas orthologs of *cje0566* and *cje1441* genes were detected in 22% and 14% of the genomes, respectively. Only 13% (353 of 2791) of the genomes contained more than a single DNase gene (Fig. 1), and only 25 genomes (0.9%) contained orthologs of all three DNase genes (Table 2, S1 Table).

**Table 2:** Distribution of eDNase genes *dns*, *cje0566* and *cje1441* in *C*. *jejuni* lineages.

**Clonal complex** ^a^	**Total** ^b^	***dns*** ^+^	***cje0566*** ^+^	***cje1441*** ^+^	**Negative**
ST-21	**764**	335 (44%)	73 (10%)	247 (32%)	260 (34%)
ST-22	**52**	14 (27%)	3 (11%)	1 (4%)	36 (64%)
ST-42	**50**	16 (32%)	3 (6%)	3 (6%)	32 (64%)
ST-45	**169**	42 (25%)	18 (11%)	2 (1%)	109 (64%)
ST-48	**201**	72 (36%)	3 (1%)	1 (0.5%)	125 (62%)
ST-52	**51**	15 (29%)	21 (41%)	2 (4%)	17 (33%)
ST-61	**70**	2 (3%)	23 (33%)	1 (1%)	44 (63%)
ST-206	**157**	21 (13%)	67 (43%)	6 (4%)	69 (44%)
ST-257	**204**	51 (25%)	151 (74%)	1 (0.5%)	34 (17%)
ST-283	**38**	0 (0%)	3 (8%)	0 (0%)	35 (92%)
ST-353	**166**	113 (68%)	16 (10%)	8 (5%)	44 (27%)
ST-354	**111**	94 (85%)	55 (50%)	19 (17%)	9 (8%)
ST-443	**100**	59 (60%)	5 (5%)	1 (1%)	39 (39%)
ST-464	**195**	40 (21%)	10 (5%)	8 (4%)	145 (74%)
ST-573	**19**	19 (100%)	19 (100%)	17 (89%)	0 (0%)
ST-574	**62**	15 (24%)	11 (18%)	49 (79%)	8 (13%)
ST-658	**59**	21 (35%)	8 (13%)	1 (2%)	35 (59%)
no^c^	**155**	54 (35%)	58 (37%)	25 (16%)	48 (31%)
other^c^	**168**	47 (28%)	59 (35%)	9 (5%)	72 (43%)
**Total**	**2791**	**1033 (37%)**	**608 (22%)**	**403 (14%)**	**1164 (42%)**

a. MLST clonal complex definitions were obtained from http://pubmlst.org/campylobacter

b. Number of draft and complete genome sequences obtained from published studies [47, 48] and draft genome sequences deposited in NCBI and pubMLST [44, 49]. Isolate names, MLST-sequence type and clonal complex, source and accession details are listed in S1 Table.

c. Other clonal complexes represented are ST-49, 179, 362, 403, 433, 446, 460, 508, 607, 661, 677, 692, 702, 1034, 1275, 1287, and 1332.

We also investigated whether the presence or absence of eDNase genes was associated with specific multi-locus sequence typing (MLST) clonal complexes [50]. Of the major MLST genotypes, the *dns* gene was proportionally overrepresented in clonal complexes ST-353, ST-354, ST-443 and ST-573, whereas the *cje0566* gene was found more in ST-257, ST-354 and ST-573, and *cje1441* gene in ST-21, ST-573 and ST-574 (Table 2, S1 Table). Of the 25 genomes positive for all three eDNase genes, the majority (17/25) was of clonal complex ST-573. Some of the major MLST genotypes had none or relatively few isolates with DNase genes, such as ST-464, ST-283, ST-42 and ST-45. Most of these MLST-types are found within agricultural environments involved in food production [50], suggesting that genetic background and shared environments may play a role in transfer of the DNase gene-containing insertion elements.

### *C*. *jejuni* strain RM1221 is unable to form a biofilm during static incubation

**Fig 2:**
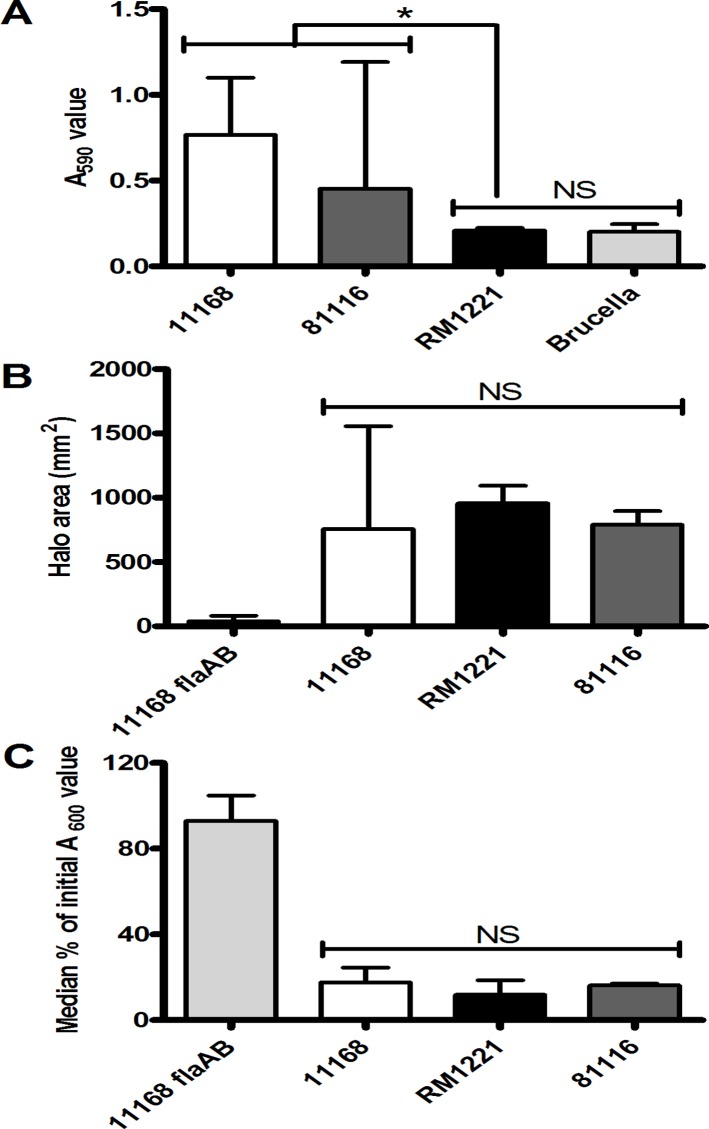
Strain RM1221 is unable to form a monospecies biofilm but exhibits both swarming and autoagglutination (AAG). Biofilm formation (A) of RM1221 (light grey bars) was measured by crystal violet staining and compared to NCTC 11168 (white bars), 81116 (dark grey bars), and a test tube containing only Brucella medium (black bar). Swarming ability (B) was calculated by measuring halo area on soft agar after 48 hours incubation in microaerobic conditions. Autoagglutination assessment (C) was carried out by observing the reduction in A_600_ measurement over a 24 hour period. Both B and C show data for 11168 (white bars), RM1221 (light grey bars), 81116 (dark grey bars) and 11168 *ΔflaAB* (dark grey bars). Bars represent the median, error bars show range and significance was measured using Mann-Whitney tests (* = *P<0*.*05*).

We selected *C*. *jejuni* strain RM1221 to further investigate the potential role of eDNase genes in biofilm formation, as it is one of the three isolates containing all three investigated eDNase genes. In previous studies investigating the role of chicken juice on biofilm formation by *C*. *jejuni* [39], we observed that chicken isolate RM1221 formed a poor biofilm in Brucella media alone. We confirmed this by comparing biofilm formation by *C*. *jejuni* strains NCTC 11168, 81116 and RM1221 using crystal violet staining, as there is a clear difference between the levels of biofilm formation of strains NCTC 11168 and 81116 versus strain RM1221, as the latter showed very little difference to the negative control (Brucella media only) (Fig. 2). Analysis by light microscopy showed that although RM1221 cells display initial attachment to the glass surface, this does not progress to the development of microcolonies (S1 Fig), unlike strains NCTC 11168 and 81116 [35, 39]. Assessment of cell viability showed that there was no difference in viability between strains RM1221, NCTC 11168 and 81116 following static culture for up to 48 hours (S1 Fig). Strain RM1221 showed comparable levels of motility to NCTC 11168 in broth cultures, suggesting that the lack of biofilm formation was not due to reduced motility or absence of flagella [33, 35]. The absence of biofilm formation by RM1221 was also not due to differences in growth in shaking cultures, nor chemotactic motility as measured by swarming, or autoagglutination, which were all comparable to strain NCTC 11168 (Fig. 2) and significantly higher than that of an aflaggelated mutant of NCTC 11168 (Δ*flaAB*).

### *C*. *jejuni* RM1221 is able to degrade pre-existing biofilms of other *C*. *jejuni* strains

**Fig 3:**
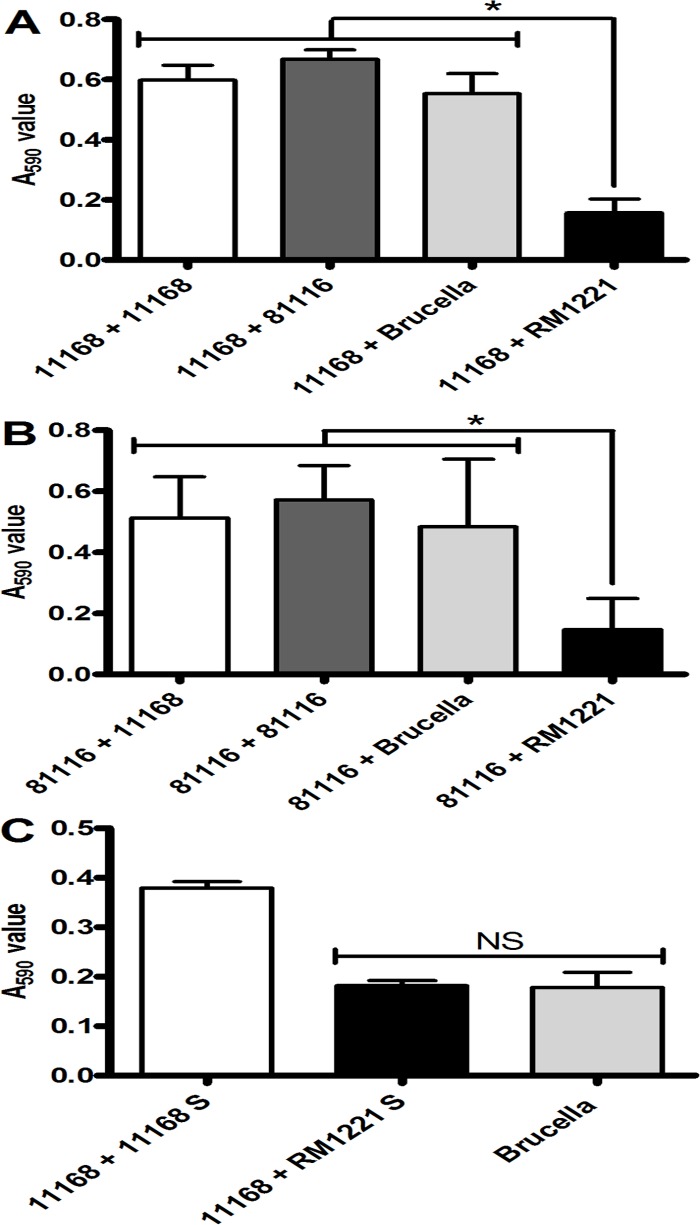
Co-incubation of pre-formed biofilms with RM1221 leads to biofilm degradation. Biofilms of NCTC 11168 (A and D) and 81116 (B) were allowed to form in static aerobic conditions for 24 hours before a further 24 hour treatment with RM1221 cell culture (A and B), or the cell free spent media of RM1221 (C). Graphs A, B and C show median A_590_ values of each treatment. Bars represent the median, error bars show range and significance was measured using Mann-Whitney tests (* = *P<0*.*05*).

We subsequently investigated whether the factors inhibiting biofilm formation by strain RM1221 are also able to affect biofilm formation of other *C*. *jejuni* strains. To test this, we grew biofilms of *C*. *jejuni* NCTC 11168 and 81116 for 24 h, and then incubated them for a further 24 h with fresh media containing either biofilm forming strains (NCTC 11168 or 81116) or strain RM1221. Fresh Brucella media was used as a negative control. Replacement with either fresh medium or medium containing 81116 or NCTC 11168 had two consequences: biofilm at the primary air-surface interface was enhanced, and a new biofilm formed at the new air-surface interface (Fig. 3). Biofilm levels were significantly reduced in tubes containing *C*. *jejuni* RM1221 in the secondary culture (Fig. 3), suggesting that not only is RM1221 a poor biofilm forming strain, but the presence of viable *C*. *jejuni* RM1221 can degrade a pre-existing *Campylobacter* biofilm.

To assess whether the negative effects of strain RM1221 on biofilm levels is due to the presence of the cells or an extracellular factor, cell-free media was prepared from *C*. *jejuni* RM1221 cultures grown under microaerobic conditions at 37°C overnight (see Materials and Methods). Cell-free supernatant from *C*. *jejuni* RM1221 culture was added to a 24 h biofilm culture of *C*. *jejuni* NCTC 11168, and this resulted in degradation of the biofilm to background levels (Fig. 3). As a control, cell-free supernatant from a *C*. *jejuni* NCTC 11168 culture did not affect biofilm formation. This suggests that the factor disrupting biofilm formation is soluble in spent media and is either actively secreted, results from cell lysis, or is a metabolic by-product.

### Disruption of *cje1441* restores biofilm formation and abolishes degradation of existing biofilms

One of the major differences between strains NCTC 11168, 81116 and RM1221 is the presence of the CJIE1-CJIE4 insertion elements (Fig. 1), of which each contain secreted proteins and secretion systems [15, 16, 51]. Since biofilms of *C*. *jejuni* strain 81–176 contain eDNA and are enhanced by the addition of exogenous DNA [33], we hypothesised that the ability of RM1221 to degrade biofilm is the result of a secreted DNase (*cje0256* (*dns*), *cje0566*, or *cje1441*). Like strain 81–176, eDNA can be observed in a mature biofilm of strain NCTC 11168 (S2 Fig). We were able to inactivate the *cje1441* gene in strain RM1221 by insertion of an antibiotic resistance cassette (see Materials and Methods). We were not able to inactive the *dns* or *cje0566* genes despite repeated attempts, which confirms the proposed role of *dns* and *cje0566* in preventing natural competence [15, 16]. Likewise, we were unable to complement the *cje1441* mutation, as constructs expressing the eDNase genes from a constitutive promoter invariably accrued spontaneous promoter mutations, suggesting that expression of *C*. *jejuni* eDNase genes in *E*. *coli* is toxic.

**Fig 4:**
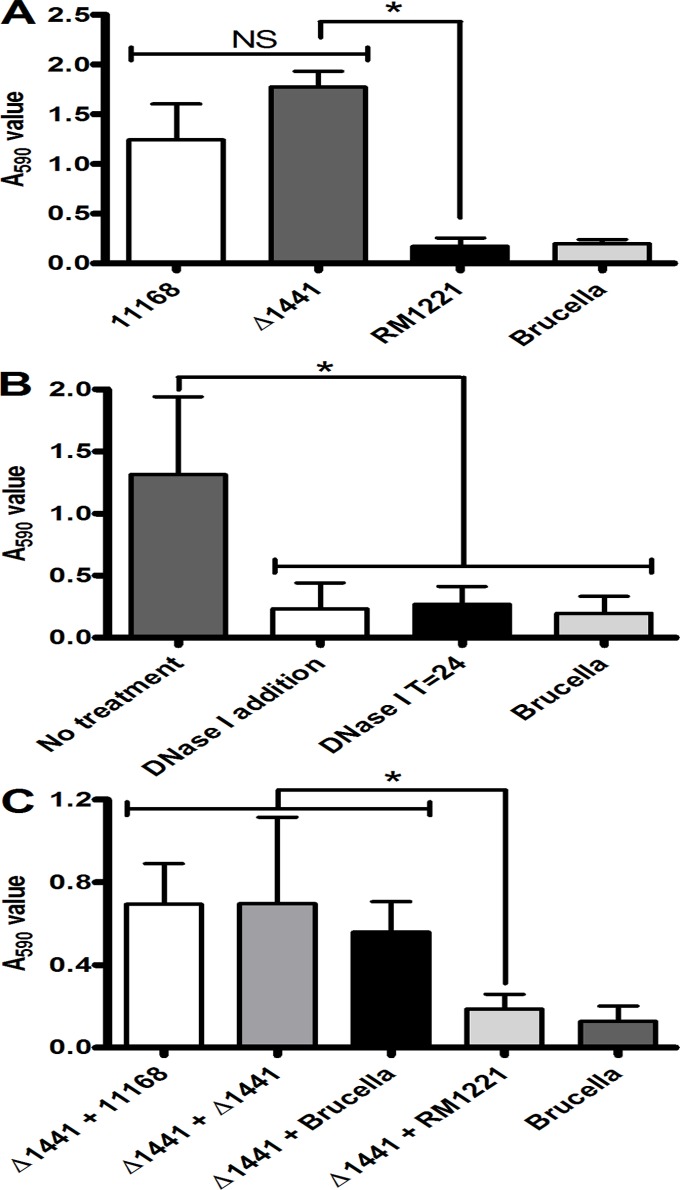
Inactivation of the *cje1441* eDNase gene restores biofilm formation by *C*. *jejuni* strain RM1221. (A) shows biofilm formation of NCTC 11168 (white bar), Δ*1441* (dark grey bar), RM1221 (black bar) and a Brucella medium only control (light grey bar). The Δ*1441* mutant shows similar levels of biofilm formation to NCTC 11168 and a significant increase in biofilm formation compared to the parent strain RM1221. (B) Shows that the biofilm produced by the Δ*1441* mutant is susceptible to degradation by DNase I (white bar) and leads to levels of staining indistinguishable from the Brucella medium only control (black bars). (C) Shows biofilm formation of the Δ*1441* mutant following secondary co-culture with strain NCTC 11168 (white bars), the Δ*1441* mutant (dark grey bars), Brucella medium (black bars), or the RM1221 parent strain (light grey bars) showing that deletion of *cje1441* inhibits the biofilm degrading ability of RM1221. Bars represent the median, error bars show range and significance was measured using Mann-Whitney tests (* = *P<0*.*05*).

Inactivation of the *cje1441* gene in RM1221 resulted in a significant increase in biofilm formation when compared to wild type RM1221, and produced similar levels of biofilm when compared to strain NCTC 11168 (Fig. 4). The level of biofilm eDNA of the Δ*1441* mutant was comparable to stain NCTC 11168 (S2 Fig). Strain RM1221 did not form a biofilm and eDNA was not detected in these assays. Inactivation of *cje1441* did not affect chemotactic motility, nor did it affect autoagglutination or growth (S3 Fig), suggesting that motility and flagellar expression were comparable to that of the parental wild-type strain RM1221. Biofilms formed by the Δ*1441* mutant were sensitive to DNase I treatment (Fig. 4), supporting the observation that DNA is present in the ECM of the Δ*1441* mutant and contributing to the biofilm structure. Furthermore, we observed that co-culture of strain NCTC 11168 and the Δ*1441* mutant resulted in biofilm levels similar to those observed with NCTC 11168 alone, thus *cje1441* contributes to the ability of RM1221 to degrade pre-formed *C*. *jejuni* biofilms (Fig. 4).

### *C*. *jejuni* RM1221 is able to degrade exogenous DNA

**Fig 5:**
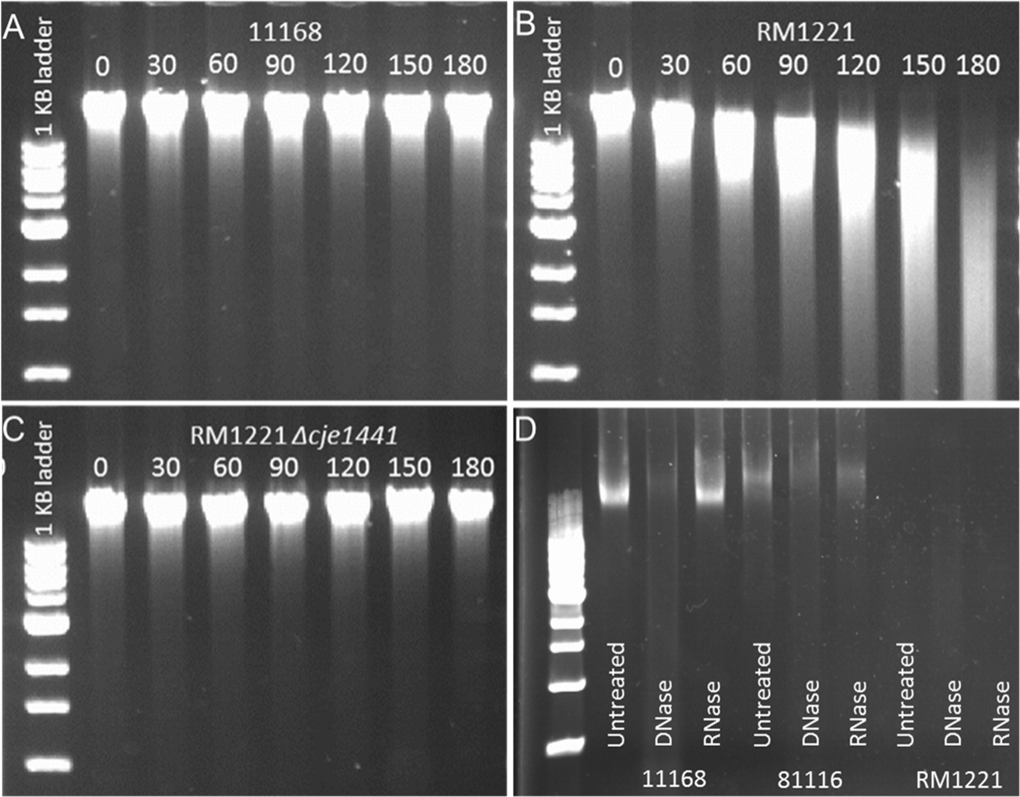
*C*. *jejuni* RM1221 is able to degrade DNA in both static and shaking suspensions. The ability of NCTC 11168 (A), RM1221 (B) and the Δ*1441* mutant (C) to degrade NCTC 11168 genomic DNA was assessed by incubation of cell suspensions with genomic DNA at 37°C for three hours. Both NCTC 11168 and the Δ*1441* mutant are unable to degrade the genomic DNA, with a band of genomic DNA of >10 kb remaining for the duration of the assay, while incubation with RM1221 results in degradation of genomic DNA (B), indicated by the ‘smearing’ shown as the time course progresses. RM1221 overnight suspensions were also shown to contain no eDNA when compared to NCTC 11168 and 81116 (D), again indicating that RM1221 is able to degrade its own exogenous DNA.

To demonstrate the DNA-specific activity of the *C*. *jejuni* RM1221 eDNases, we mixed genomic DNA from *C*. *jejuni* strain NCTC 11168 with washed RM1221 cells, and incubated this mixture at 37°C. No DNA degradation was observed when *C*, *jejuni* strain NCTC 11168 was mixed with genomic DNA (Fig. 5). However, addition of *C*. *jejuni* RM1221 resulted in time-dependent degradation of *C*. *jejuni* genomic DNA over a three hour time course (Fig. 5). DNA degradation was abolished in the *C*. *jejuni* Δ*1441* mutant, suggesting that the CJE1441 eDNase makes an important contribution to the DNA degradation observed in the parental RM1221 strain (Fig. 5). We also tested whether RM1221 was able to degrade its own DNA, to exclude a role for DNA methylation. As with the NCTC 11168 genomic DNA, RM1221 genomic DNA was rapidly degraded, indicating that the DNase activity is non-specific (data not shown). Cell suspensions of RM1221 could also degrade linear PCR fragments and uncut plasmid DNA, both methylated and non-methylated (S4 Fig). This DNase activity was inhibited in the presence of EDTA. This DNase activity was not detected in strain NCTC 11168 or the Δ*1441* mutant.

Analysis of levels of extracellular DNA purified from overnight growth cultures of *C*. *jejuni* NCTC 11168, 81116 and RM1221 showed the presence of high molecular weight nucleic acids, running with the same mobility as genomic DNA, in the supernatants from strains NCTC 11168 and 81116 but not for RM1221 (Fig. 5). These fragments were sensitive to DNase I digestion but not RNase A treatment, suggesting that they are high molecular weight DNA molecules. As with previous experiments, we did not observe any significant differences in cell viability or growth, suggesting that variations in eDNA release between the *C*. *jejuni* strains is not caused by variations in viability (i.e. cell death and lysis) or rate of growth (data not shown).

Taken together these results suggest that *C*. *jejuni* NCTC 11168 releases DNA during growth, and that this DNA contributes to biofilm formation. RM1221 can degrade this DNA and thus disrupt both *de novo* biofilm formation and pre-formed biofilm. This activity is highly dependent on the CJE1441 eDNase, expressed from the *cje1441* gene on the insertion element CJIE4. This DNase is likely an endonuclease that is dependent on metal ions for activity.

## Discussion

Biofilms play an important role in the lifestyle of many bacteria, and cause both considerable problems in healthcare and the food industry. One problematic aspect of biofilm formation is its contribution to transmission and survival of bacterial and fungal pathogens. There is also now an increasing body of evidence that suggests biofilms may assist in C. *jejuni* food chain persistence [24, 39, 52] and recent work has shown that eDNA is important in the maturation of *C*. *jejuni* strain 81–176 biofilms [33]. A better understanding of the mechanisms involved in biofilm formation by *C*. *jejuni* could lead to development of applications targeting *C*. *jejuni* transmission in the food chain. In this study, we have shown that there are differences in biofilm formation between three *C*. *jejuni* reference isolates, and have shown that eDNase activity results in degradation of pre-formed *C*. *jejuni* biofilms, as well as prevention of *de novo* biofilm formation. This work highlights how naturally-occurring eDNase activity may be able to weaken or destroy natural biofilms, e.g. in food processing environments.

Most *C*. *jejuni* isolates are naturally competent, and readily take up DNA from the environment and in some cases, recombine this into their genome. One of the consequences is that *C*. *jejuni* shows a high level of genetic diversity, both at the sequence level and at the level of genetic content [29, 53]. In this study we have used *C*. *jejuni* reference strain RM1221, which is not naturally transformable due to the expression of three eDNase genes from the CJIE1, CJIE2 and CJIE4 insertion elements [15, 16]. Although the biological function of the eDNase activity in *C*. *jejuni* is yet to be elucidated, it is possible that it protects isolates with the insertion element against allelic exchange with insertion element-negative flanking sequences, as this incurs the risk of losing the insertion element, which offer some evolutionary advantage. Our investigation of a large (N = 2791) collection of *C*. *jejuni* genome sequences showed that the eDNase genes are differentially distributed in *C*. *jejuni*, and that very few genomes contain three copies of an eDNase gene. However, with 58% of the *C*. *jejuni* genomes included being positive for at least one eDNase gene, this suggests that there will be eDNase-expressing isolates present in many agricultural environments, and these may have a profound effect on biofilms produced or colonised.

We here also show that the expression of eDNase activity has another consequence, severely reducing biofilm formation by *C*. *jejuni* strain RM1221. Further support for a role of the eDNases in restricting biofilm formation was obtained by inactivation of the *cje1441* eDNase gene in strain RM1221, which allowed RM1221 to form biofilms. The eDNase activity and lack of natural competence has so far precluded robust genetic manipulation of strain RM1221 (other than conjugation via tri-parental mating [41]), and our successful inactivation of *cje1441* is to our knowledge the first genetic manipulation of the RM1221 chromosome. The eDNase genes pose technical problems for genetic manipulation and cloning, as their intracellular expression can lead to cytoplasmic DNase activity and cell death, thus hampering cloning and expression in *E*. *coli*. This has also been reported for eDNase proteins of other bacteria, such as the eDNase proteins from the predatory bacterium *Bdellovibrio bacteriovorus*, where expression was found to be lethal in *E*. *coli* [14]. Similarly, expression of active DNase I by *E*. *coli* could only be achieved by the use of the very tightly controlled expression plasmid pDOC55 [54]. Such plasmids are not available for *C*. *jejuni*, and hence genetic manipulation of the eDNase genes in *C*. *jejuni* is technically challenging.

The importance of eDNA in bacterial biofilms is now well recognised [4, 33], and has attracted attention as a target for enzymatic or chemical treatment for disinfection purposes. DNase I is effective in interfering with the biofilms of the foodborne pathogens *Listeria monocytogenes* [55] and of *E*. *coli* [56], but also mixed species biofilms. Biofilms found in activated sludge flocs have eDNA from lysed cells forming close interactions with the viable cells within the biofilm [57], and affected microcolony formation within the biofilm. Similarly, mixed biofilms with *Staphylococcus epidermidis* and *Candida albicans* are also affected by DNase treatment [58], suggesting that DNase is able to modify both mixed species and mixed kingdom biofilms. Addition of exogenous DNase is effective in reducing biofilms of pathogenic bacteria, such *Neisseria gonorrhoeae* [59], *Garderella vaginalis* [6] and *L*. *monocytogenes* [60].

Many species which form biofilms are also able to produce and export extracellular DNase proteins, and eDNase proteins have multiple functions. The *P*. *aeruginosa* eDNase PA3909 is involved in DNA degradation, providing an additional nutrient source, and its expression is induced in phosphate limiting conditions [13], whereas in *Shewanella oneidensis*, expression of the nucleases ExeM and ExeS is strongly induced if DNA is the sole nutrient source, and deletion of the ExeM gene leads to a significantly reduced growth rate [61]. Finally, the eDNase genes of *Staphylococcus aureus* are involved in immune evasion, and their expression during host infection aids the escape of *S*. *aureus* from the DNA ‘nets’ which are secreted by neutrophils [8]. In the case of *C*. *jejuni* [15, 16], and other bacteria such as *Vibrio cholerae* [62], the eDNase proteins restrict natural transformation.

Many bacteria which produce eDNase enzymes are still able to form biofilms and appear to utilise the enzymes in order to modify their biofilm structures. Two well-studied examples of eDNase-positive bacterial species that can form biofilms are *V*. *cholerea* [11] and *S*. *aureus* [63]. Since the eDNase genes of RM1221 are classified as non-specific DNA/RNA endonucleases, they are not expected to have stringent specificity regarding the source, methylation or sequence of the DNA targeted for digestion, and this was confirmed by absence of eDNA in RM1221 cultures (Fig. 5). We hypothesize that rapid degradation of eDNA restricts its deposition on surfaces, and as such inhibits the initial stages of attachment of *C*. *jejuni* to these surfaces [39].

It is important to note that not all bacterial species show reduction of biofilm formation following treatment with DNases. When the opportunistic pathogen *Burkholderia cenocepacia* was exposed to DNase it produced significantly denser biofilms [64], while *Helicobacter pylori* biofilms remain unaffected following treatment with DNase I [65]. Finally, the presence of eDNA on a surface inhibits biofilm formation by *Salmonella enterica* serovars Typhimurium and Typhi [66]. These examples show that DNase treatment may not be effective in the case of all single species biofilms. However many naturally occurring biofilms, such as are found in processing plants, are comprised of multiple species and so DNase treatment should still be considered an effective mechanism of at least partially degrading biofilms and allowing better penetration of antimicrobials.

Treatment of biofilm-based bacterial infections with DNases has increased in recent years. Impregnation of the biomaterial polymethylmethacrylate with DNase I lead to reduced adherence of *P*. *aeruginosa* and *S*. *aureus*, without a detrimental effect on adhesion and proliferation of human cells [67]. Human recombinant DNase dornase alpha (brand name Pulmozyme) is frequently used in the treatment of cystic fibrosis [68], and it also degrades DNA within biofilms isolated from children with recurrent acute otitis media [69]. DNase I treatment has also been shown to reduce established *Bordetella bronchiseptica* and *B*. *pertussis* biofilms from the mouse respiratory tract [70]. *In vitro* treatment of biofilms of non-typeable *Haemophilus influenzae* with DNase I also allowed increased bacterial killing by β defensins [71], this suggests that even in biofilms where DNase I treatment does not have a direct biofilm reducing effect it can still be a useful addition to a treatment regimen.

Treatment with DNase enzymes is becoming a common intervention in treatment of some biofilm infections and chronic conditions such as cystic fibrosis, but DNase production is costly. This is not considered problematic within the medical industry, but the high cost of production severely limits its potential for use in the food chain. Within the food industry, the use of naturally produced bacterial eDNases could be a suitable alternative to DNase I use. Bacteria such as *Aeromonas* sp. produce several secreted DNase enzymes [72] and in species such as *Streptococcus agalactiae*, some of these eDNase proteins are heat stable [73]. Many of the DNase-positive bacteria have low complexity growth requirements and do not have the ethical or legal issues, which may preclude or limit the use of DNase obtained from animals, or recombinant products from genetically modified organisms. The cell-free extracts of *C*. *jejuni* RM1221 retain their DNase activity, and are able to degrade *C*. *jejuni* biofilms even after a ten minute heat treatment (data not shown). This suggests that the eDNase enzymes of RM1221 are relatively heat stable and could potentially be a source of easily obtainable DNase proteins for use during food chain cleaning, although such an application requires further consideration and investigation to ensure that any supernatant derived products is safe for use, particularly from pathogenic bacteria.

In conclusion, eDNase activity inhibits biofilm formation by *C*. *jejuni* RM1221, and this eDNase activity can be utilised to degrade biofilms formed by other *C*. *jejuni* strains, using either live RM1221 cells or cell-free supernatant. Since DNase treatment has been proved to be so effective against both bacterial and fungal biofilms, extraction of eDNase enzymes from *C*. *jejuni* strains such as RM1221 could in future provide a cost effective alternative source of DNase enzymes, and assist in developing applications improving food safety by prevention of biofilm-assisted transmission of foodborne pathogens such as *C*. *jejuni*.

## Supporting Information

S1 Fig *C*. *jejuni* strain RM1221 is unable to form microcolonies or biofilms. (A) and (B) show representative images of the air/liquid interface of a glass slide following 48 hours of static incubation at 37°C in aerobic conditions. (A) shows a slide incubated with RM1221 cells and (B) shows a slide incubated with NCTC 11168. The highlighted area in (A) shows potentially attached RM1221 cells, although no progression to microcolony formation is observed. (C) shows representative images of spot plates following 48 hour static incubation at 37°C in aerobic conditions.
(TIF)

S2 Fig Extracellular DNA is present in *C*. *jejuni* biofilms. (A) Representative image of Green fluorescent protein (GFP)-expressing NCTC 11168 biofilms (strain NCTC 11168 GFP^+^, see Table 1) counter stained with DAPI. A diffuse blue dye can be seen around the GFP-expressing cells suggesting that there is a large quantity of eDNA present within the mature biofilm. (B) Three biological replicates showing ethidium bromide-stained DNA isolated from biofilm samples from strains NCTC 11168, RM1221, and Δ*1441* after agarose gel electrophoresis.
(TIF)

S3 Fig *C*. *jejuni* RM1221 Δ*1441* and its parent strain show no significant difference in swarming, autoagglutination or growth. *C*. *jejuni* strains NCTC 11168 (white), its non-motile *ΔflaAB* mutant (dark grey), RM1221 (black bars) and the Δ*1441* mutant (light grey) were compared for their ability to swarm (A) and autoagglutinate (B). In both tests no statistical difference was observed between Δ*1441* and the wild-type. Panel C shows growth over a 24 hour period for Δ*1441* (light grey triangles), RM1221 wild-type (black circles) and NCTC 11168 (white squares). Bars represent the median, error bars show range and significance was measured using Mann-Whitney tests.
(TIF)

S4 Fig *C*. *jejuni* RM1221 cell suspension has EDTA-dependent endonuclease (DNase) activity. Plasmid DNA (70 ng) was incubated with cell suspensions for 60 minutes at 37°C prior to agarose gel electrophoresis. Plasmid DNA is almost entirely degraded in reactions containing RM1221 cell suspension, but not NCTC 11168 or Δ*1441*.
(TIF)

S1 Table Presence/absence analysis for homologs of the *cje0256*, *cje0566* and *cje1441* eDNase genes in *C*. *jejuni*. (XLSX)

## References

[1] Donlan RM. Biofilms: microbial life on surfaces. Emerg Infect Dis 2002;8: 881–890. 12194761 10.3201/eid0809.020063 PMC2732559

[2] McCrate OA, Zhou X, Reichhardt C, Cegelski L. Sum of the parts: composition and architecture of the bacterial extracellular matrix. J Mol Biol 2013;425: 4286–4294. 10.1016/j.jmb.2013.06.022 23827139 PMC3812305

[3] Flemming HC, Wingender J. The biofilm matrix. Nat Rev Microbiol 2010;8: 623–633. 10.1038/nrmicro2415 20676145

[4] Whitchurch CB, Tolker-Nielsen T, Ragas PC, Mattick JS. Extracellular DNA required for bacterial biofilm formation. Science 2002;295: 1487 11859186 10.1126/science.295.5559.1487

[5] Mann EE, Rice KC, Boles BR, Endres JL, Ranjit D, Chandramohan L, et al Modulation of eDNA Release and Degradation Affects *Staphylococcus aureus* Biofilm Maturation. PLoS One 2009;4: e5822 10.1371/journal.pone.0005822 19513119 PMC2688759

[6] Hymes SR, Randis TM, Sun TY, Ratner AJ. DNase inhibits *Gardnerella vaginalis* biofilms in vitro and in vivo. J Infect Dis 2013;207: 1491–1497. 10.1093/infdis/jit047 23431033 PMC3627197

[7] Jakubovics NS, Shields RC, Rajarajan N, Burgess JG. Life after death: the critical role of extracellular DNA in microbial biofilms. Lett Appl Microbiol 2013;57: 467–475. 10.1111/lam.12134 23848166

[8] Berends ET, Horswill AR, Haste NM, Monestier M, Nizet V, von Kockritz-Blickwede M. Nuclease expression by *Staphylococcus aureus* facilitates escape from neutrophil extracellular traps. J Innate Immun 2010;2: 576–586. 10.1159/000319909 20829609 PMC2982853

[9] Riyapa D, Buddhisa S, Korbsrisate S, Cuccui J, Wren BW, Stevens MP, et al Neutrophil extracellular traps exhibit antibacterial activity against *Burkholderia pseudomallei* and are influenced by bacterial and host factors. Infect Immun 2012;80: 3921–3929. 10.1128/IAI.00806-12 22927051 PMC3486034

[10] Beenken KE, Spencer H, Griffin LM, Smeltzer MS. Impact of Extracellular Nuclease Production on the Biofilm Phenotype of *Staphylococcus aureus* under In Vitro and In Vivo Conditions. Infect Immun 2012;80: 1634–1638. 10.1128/IAI.06134-11 22354028 PMC3347440

[11] Seper A, Fengler VH, Roier S, Wolinski H, Kohlwein SD, Bishop AL, et al Extracellular nucleases and extracellular DNA play important roles in *Vibrio cholerae* biofilm formation. Mol Microbiol 2011;82: 1015–1037. 10.1111/j.1365-2958.2011.07867.x 22032623 PMC3212620

[12] Finkel SE, Kolter R. DNA as a Nutrient: Novel Role for Bacterial Competence Gene Homologs. J Bacteriol 2001;183: 6288–6293. 11591672 10.1128/JB.183.21.6288-6293.2001 PMC100116

[13] Mulcahy H, Charron-Mazenod L, Lewenza S. *Pseudomonas aeruginosa* produces an extracellular deoxyribonuclease that is required for utilization of DNA as a nutrient source. Environ Microbiol 2010;12: 1621–1629. 10.1111/j.1462-2920.2010.02208.x 20370819

[14] Lambert C, Sockett RE. Nucleases in *Bdellovibrio bacteriovorus* contribute towards efficient self-biofilm formation and eradication of preformed prey biofilms. FEMS Microbiol Lett 2013;340: 109–116. 10.1111/1574-6968.12075 23297829 PMC3593177

[15] Gaasbeek EJ, Wagenaar JA, Guilhabert MR, van Putten JP, Parker CT, van der Wal FJ. Nucleases encoded by the integrated elements CJIE2 and CJIE4 inhibit natural transformation of *Campylobacter jejuni* . J Bacteriol 2010;192: 936–941. 10.1128/JB.00867-09 20023031 PMC2812962

[16] Gaasbeek EJ, Wagenaar JA, Guilhabert MR, Wosten MM, van Putten JP, van der Graaf-van Bloois L, et al A DNase encoded by integrated element CJIE1 inhibits natural transformation of *Campylobacter jejuni* . J Bacteriol 2009;191: 2296–2306. 10.1128/JB.01430-08 19151136 PMC2655492

[17] Tam CC, Rodrigues LC, Viviani L, Dodds JP, Evans MR, Hunter PR, et al Longitudinal study of infectious intestinal disease in the UK (IID2 study): incidence in the community and presenting to general practice. Gut 2012;61: 69–77. 10.1136/gut.2011.238386 21708822 PMC3230829

[18] Zautner AE, Johann C, Strubel A, Busse C, Tareen AM, Masanta WO, et al Seroprevalence of campylobacteriosis and relevant post-infectious sequelae. Eur J Clin Microbiol Infect Dis 2014;33: 1019–1027. 10.1007/s10096-013-2040-4 24413899 PMC4013439

[19] Bronowski C, James CE, Winstanley C. Role of environmental survival in transmission of *Campylobacter jejuni* . FEMS Microbiol Lett 2014;356: 8–19. 10.1111/1574-6968.12488 24888326

[20] Joshua GW, Guthrie-Irons C, Karlyshev AV, Wren BW. Biofilm formation in *Campylobacter jejuni* . Microbiology 2006;152: 387–396. 16436427 10.1099/mic.0.28358-0

[21] Sulaeman S, Hernould M, Schaumann A, Coquet L, Bolla JM, De E, et al Enhanced adhesion of *Campylobacter jejuni* to abiotic surfaces is mediated by membrane proteins in oxygen-enriched conditions. PloS One 2012;7: e46402 10.1371/journal.pone.0046402 23029510 PMC3460892

[22] Guyard-Nicodeme M, Tresse O, Houard E, Jugiau F, Courtillon C, El Manaa K, et al Characterization of *Campylobacter* spp. transferred from naturally contaminated chicken legs to cooked chicken slices via a cutting board. Int J Food Microbiol 2013;164: 7–14. 10.1016/j.ijfoodmicro.2013.03.009 23587707

[23] Gunther NW, Chen CY. The biofilm forming potential of bacterial species in the genus *Campylobacter* . Food Microbiol 2009;26: 44–51. 10.1016/j.fm.2008.07.012 19028304

[24] Balamurugan S, Nattress FM, Baker LP, Dilts BD. Survival of *Campylobacter jejuni* on beef and pork under vacuum packaged and retail storage conditions: examination of the role of natural meat microflora on *C*. *jejuni* survival. Food Microbiol 2011;28: 1003–1010. 10.1016/j.fm.2011.01.012 21569945

[25] Teh KH, Flint S, French N. Biofilm formation by *Campylobacter jejuni* in controlled mixed-microbial populations. Int J Food Microbiol 2010;143: 118–124. 10.1016/j.ijfoodmicro.2010.07.037 20805009

[26] Kalmokoff M, Lanthier P, Tremblay TL, Foss M, Lau PC, Sanders G, et al Proteomic analysis of *Campylobacter jejuni* 11168 biofilms reveals a role for the motility complex in biofilm formation. J Bacteriol 2006;188: 4312–4320. 16740937 10.1128/JB.01975-05 PMC1482957

[27] Dingle KE, Colles FM, Wareing DRA, Ure R, Fox AJ, Bolton FE, et al Multilocus Sequence Typing System for *Campylobacter jejuni* . J Clin Microbiol 2001;39: 14–23. 11136741 10.1128/JCM.39.1.14-23.2001 PMC87672

[28] Fouts DE, Mongodin EF, Mandrell RE, Miller WG, Rasko DA, Ravel J, et al Major Structural Differences and Novel Potential Virulence Mechanisms from the Genomes of Multiple *Campylobacter* Species. PLoS Biol 2005;3: e15 15660156 10.1371/journal.pbio.0030015 PMC539331

[29] Parker CT, Quinones B, Miller WG, Horn ST, Mandrell RE. Comparative genomic analysis of *Campylobacter jejuni* strains reveals diversity due to genomic elements similar to those present in *C*. *jejuni* strain RM1221. J Clin Microbiol 2006;44: 4125–4135. 16943349 10.1128/JCM.01231-06 PMC1698300

[30] Svensson SL, Davis LM, MacKichan JK, Allan BJ, Pajaniappan M, Thompson SA, et al The CprS sensor kinase of the zoonotic pathogen *Campylobacter jejuni* influences biofilm formation and is required for optimal chick colonization. Mol Microbiol 2009;71: 253–272. 10.1111/j.1365-2958.2008.06534.x 19017270 PMC2771394

[31] Frirdich E, Vermeulen J, Biboy J, Soares F, Taveirne ME, Johnson JG, et al Peptidoglycan LD-carboxypeptidase Pgp2 influences *Campylobacter jejuni* helical cell shape and pathogenic properties and provides the substrate for the DL-carboxypeptidase Pgp1. J Biol Chem 2014;289: 8007–8018. 10.1074/jbc.M113.491829 24394413 PMC3961634

[32] Oh E, Jeon B. Role of alkyl hydroperoxide reductase (AhpC) in the biofilm formation of *Campylobacter jejuni* . PLoS One 2014;9: e87312 10.1371/journal.pone.0087312 24498070 PMC3909096

[33] Svensson SL, Pryjma M, Gaynor EC. Flagella-Mediated Adhesion and Extracellular DNA Release Contribute to Biofilm Formation and Stress Tolerance of *Campylobacter jejuni* . PLoS One 2014;9: e106063 10.1371/journal.pone.0106063 25166748 PMC4148357

[34] Parkhill J, Wren BW, Mungall K, Ketley JM, Churcher C, Basham D, et al The genome sequence of the food-borne pathogen *Campylobacter jejuni* reveals hypervariable sequences. Nature 2000;403: 665–668. 10688204 10.1038/35001088

[35] Reuter M, Mallett A, Pearson BM, van Vliet AH. Biofilm formation by *Campylobacter jejuni* is increased under aerobic conditions. Appl Environ Microbiol 2010;76: 2122–2128. 10.1128/AEM.01878-09 20139307 PMC2849235

[36] Manning G, Duim B, Wassenaar T, Wagenaar JA, Ridley A, Newell DG. Evidence for a genetically stable strain of *Campylobacter jejuni* . Appl Environ Microbiol 2001;67: 1185–1189. 11229909 10.1128/AEM.67.3.1185-1189.2001 PMC92712

[37] Jackson ME, Pratt JM, Stoker NG, Holland IB. An inner membrane protein N-terminal signal sequence is able to promote efficient localisation of an outer membrane protein in *Escherichia coli* . EMBO J 1985;4: 2377–2383. 3908094 10.1002/j.1460-2075.1985.tb03942.x PMC554513

[38] Reuter M, van Vliet AHM. Signal balancing by the CetABC and CetZ chemoreceptors controls energy taxis in *Campylobacter jejuni* . PLoS One 2013;8: e54390 10.1371/journal.pone.0054390 23382896 PMC3558505

[39] Brown HL, Reuter M, Salt LJ, Cross KL, Betts RP, van Vliet AHM. Chicken juice enhances surface attachment and biofilm formation of *Campylobacter jejuni* . Appl Environ Microbiol 2014;80: 7053–7060. 10.1128/AEM.02614-14 25192991 PMC4249011

[40] van Vliet AHM, Wood A. C., Henderson J., Wooldridge K. G., Ketley JM (1998) Genetic Manipulation of enteric *Campylobacter* species In: Williams P., Ketley J., Salmond G, editors. Bacterial Pathogenesis. London: Academic Press pp. 407–420.

[41] Miller WG, Bates AH, Horn ST, Brandl MT, Wachtel MR, Mandrell RE. Detection on surfaces and in Caco-2 cells of *Campylobacter jejuni* cells transformed with new *gfp*, *yfp*, and *cfp* marker plasmids. Appl Environ Microbiol 2000;66: 5426–5436. 11097924 10.1128/aem.66.12.5426-5436.2000 PMC92478

[42] Donahue JP, Israel DA, Peek RM, Blaser MJ, Miller GG. Overcoming the restriction barrier to plasmid transformation of *Helicobacter pylori* . Mol Microbiol 2000;37: 1066–1074. 10972825 10.1046/j.1365-2958.2000.02036.x

[43] Golden NJ, Acheson DW. Identification of motility and autoagglutination *Campylobacter jejuni* mutants by random transposon mutagenesis. Infect Immun 2002;70: 1761–1771. 11895937 10.1128/IAI.70.4.1761-1771.2002 PMC127829

[44] Jolley KA, Maiden MC. BIGSdb: Scalable analysis of bacterial genome variation at the population level. BMC Bioinformatics 2010;11: 595 10.1186/1471-2105-11-595 21143983 PMC3004885

[45] Gillespie JJ, Wattam AR, Cammer SA, Gabbard JL, Shukla MP, Dalay O, et al PATRIC: the comprehensive bacterial bioinformatics resource with a focus on human pathogenic species. Infect Immun 2011;79: 4286–4298. 10.1128/IAI.00207-11 21896772 PMC3257917

[46] Kruczkiewicz P, Mutschall S, Barker D, Thomas J, Van Domselaar G, Gannon VPJ, et al MIST: a tool for rapid in silico generation of molecular data from bacterial genome sequences. Proc Bioinform 2013 2013: 316–323.

[47] Sheppard SK, Didelot X, Jolley KA, Darling AE, Pascoe B, Meric G, et al Progressive genome-wide introgression in agricultural *Campylobacter coli* . Mol Ecol 2013;22: 1051–1064. 10.1111/mec.12162 23279096 PMC3749442

[48] Richards VP, Lefebure T, Pavinski Bitar PD, Stanhope MJ. Comparative characterization of the virulence gene clusters (lipooligosaccharide [LOS] and capsular polysaccharide [CPS]) for *Campylobacter coli*, *Campylobacter jejuni* subsp. *jejuni* and related *Campylobacter* species. Infect Genet Evol 2013;14: 200–213. 10.1016/j.meegid.2012.12.010 23279811 PMC3622452

[49] Cody AJ, McCarthy ND, Jansen van Rensburg M, Isinkaye T, Bentley SD, Parkhill J, et al Real-time genomic epidemiological evaluation of human *Campylobacter* isolates by use of whole-genome multilocus sequence typing. J Clin Microbiol 2013;51: 2526–2534. 10.1128/JCM.00066-13 23698529 PMC3719633

[50] Colles FM, Maiden MC. *Campylobacter* sequence typing databases: applications and future prospects. Microbiology 2012;158: 2695–2709. 10.1099/mic.0.062000-0 22986295

[51] Bleumink-Pluym NM, van Alphen LB, Bouwman LI, Wosten MM, van Putten JP. Identification of a functional type VI secretion system in *Campylobacter jejuni* conferring capsule polysaccharide sensitive cytotoxicity. PLoS Pathog 2013;9: e1003393 10.1371/journal.ppat.1003393 23737749 PMC3667781

[52] Teh AH, Lee SM, Dykes GA. Do *Campylobacter jejuni* Form Biofilms in Food-Related Environments? Appl Environ Microbiol 2014;80: 5154–5160. 10.1128/AEM.01493-14 24928882 PMC4136081

[53] Clark CG, Chong PM, McCorrister SJ, Mabon P, Walker M, Westmacott GR. DNA sequence heterogeneity of *Campylobacter jejuni* CJIE4 prophages and expression of prophage genes. PLoS One 2014;9: e95349 10.1371/journal.pone.0095349 24756024 PMC3995785

[54] Worrall AF, Connolly BA. The chemical synthesis of a gene coding for bovine pancreatic DNase I and its cloning and expression in *Escherichia coli* . J Biol Chem 1990;265: 21889–21895. 2254338

[55] Harmsen M, Lappann M, Knochel S, Molin S. Role of extracellular DNA during biofilm formation by *Listeria monocytogenes* . Appl Environ Microbiol 2010;76: 2271–2279. 10.1128/AEM.02361-09 20139319 PMC2849236

[56] Zhao J, Wang Q, Li M, Heijstra BD, Wang S, Liang Q, et al *Escherichia coli* toxin gene *hipA* affects biofilm formation and DNA release. Microbiology 2013;159: 633–640. 10.1099/mic.0.063784-0 23329678

[57] Dominiak DM, Nielsen JL, Nielsen PH. Extracellular DNA is abundant and important for microcolony strength in mixed microbial biofilms. Environ Microbiol 2011;13: 710–721. 10.1111/j.1462-2920.2010.02375.x 21118344

[58] Pammi M, Liang R, Hicks J, Mistretta TA, Versalovic J. Biofilm extracellular DNA enhances mixed species biofilms of *Staphylococcus epidermidis* and *Candida albicans* . BMC Microbiol 2013;13: 257 10.1186/1471-2180-13-257 24228850 PMC3833181

[59] Zweig M, Schork S, Koerdt A, Siewering K, Sternberg C, Thormann K, et al Secreted single-stranded DNA is involved in the initial phase of biofilm formation by *Neisseria gonorrhoeae* . Environ Microbiol 2014;16: 1040–1052. 10.1111/1462-2920.12291 24119133

[60] Nguyen MH, Ojima Y, Sakka M, Sakka K, Taya M. Probing of exopolysaccharides with green fluorescence protein-labeled carbohydrate-binding module in *Escherichia coli* biofilms and flocs induced by *bcsB* overexpression. J Biosci Bioeng 2014. 10.1016/j.jbiosc.2014.03.005 24746734

[61] Godeke J, Heun M, Bubendorfer S, Paul K, Thormann KM. Roles of two *Shewanella oneidensis* MR-1 extracellular endonucleases. Appl Environ Microbiol 2011;77: 5342–5351. 10.1128/AEM.00643-11 21705528 PMC3147473

[62] Focareta T, Manning PA. Distinguishing between the extracellular DNases of *Vibrio cholerae* and development of a transformation system. Mol Microbiol 1991;5: 2547–2555. 1791765 10.1111/j.1365-2958.1991.tb02101.x

[63] Kiedrowski MR, Kavanaugh JS, Malone CL, Mootz JM, Voyich JM, Smeltzer MS, et al Nuclease modulates biofilm formation in community-associated methicillin-resistant *Staphylococcus aureus* . PLoS One 2011;6: e26714 10.1371/journal.pone.0026714 22096493 PMC3214024

[64] Novotny LA, Amer AO, Brockson ME, Goodman SD, Bakaletz LO. Structural stability of *Burkholderia cenocepacia* biofilms is reliant on eDNA structure and presence of a bacterial nucleic acid binding protein. PLoS One 2013;8: e67629 10.1371/journal.pone.0067629 23799151 PMC3682984

[65] Grande R, Di Giulio M, Bessa LJ, Di Campli E, Baffoni M, Guarnieri S, et al Extracellular DNA in *Helicobacter pylori* biofilm: a backstairs rumour. J Appl Microbiol 2011;110: 490–498. 10.1111/j.1365-2672.2010.04911.x 21143715

[66] Wang H, Huang Y, Wu S, Li Y, Ye Y, Zheng Y, et al Extracellular DNA Inhibits *Salmonella enterica* Serovar Typhimurium and *S*. *enterica* Serovar Typhi Biofilm Development on Abiotic Surfaces. Curr Microbiol 2014;68: 262–268. 10.1007/s00284-013-0468-5 24126602

[67] Swartjes JJTM, Das T, Sharifi S, Subbiahdoss G, Sharma PK, Krom BP, et al A Functional DNase I Coating to Prevent Adhesion of Bacteria and the Formation of Biofilm. Adv Funct Mater 2013;23: 2843–2849.

[68] Konstan MW, Ratjen F. Effect of dornase alfa on inflammation and lung function: potential role in the early treatment of cystic fibrosis. J Cyst Fibros 2012;11: 78–83. 10.1016/j.jcf.2011.10.003 22093951 PMC4090757

[69] Thornton RB, Wiertsema SP, Kirkham LA, Rigby PJ, Vijayasekaran S, Coates HL, et al Neutrophil extracellular traps and bacterial biofilms in middle ear effusion of children with recurrent acute otitis media—a potential treatment target. PLoS One 2013;8: e53837 10.1371/journal.pone.0053837 23393551 PMC3564866

[70] Conover MS, Mishra M, Deora R. Extracellular DNA is essential for maintaining *Bordetella* biofilm integrity on abiotic surfaces and in the upper respiratory tract of mice. PLoS One 2011;6: e16861 10.1371/journal.pone.0016861 21347299 PMC3037945

[71] Jones EA, McGillivary G, Bakaletz LO. Extracellular DNA within a nontypeable *Haemophilus influenzae*-induced biofilm binds human beta defensin-3 and reduces its antimicrobial activity. J Innate Immun 2013;5: 24–38. 10.1159/000339961 22922323 PMC3640559

[72] Pemberton JM, Kidd SP, Schmidt R. Secreted enzymes of *Aeromonas* . FEMS Microbiol Lett 1997;152: 1–10. 9228763 10.1111/j.1574-6968.1997.tb10401.x

[73] Derre-Bobillot A, Cortes-Perez NG, Yamamoto Y, Kharrat P, Couve E, Da Cunha V, et al Nuclease A (Gbs0661), an extracellular nuclease of *Streptococcus agalactiae*, attacks the neutrophil extracellular traps and is needed for full virulence. Mol Microbiol 2013;89: 518–531. 10.1111/mmi.12295 23772975

