# Supplementary figures and images for: Prevention of Biofilm Formation and Removal of Existing Biofilms by Extracellular DNases of Campylobacter jejuni
Source: PLoS One. 2015 Mar 24;10(3):e0121680. doi: 10.1371/journal.pone.0121680 (PMC4372405; doi:10.1371/journal.pone.0121680)

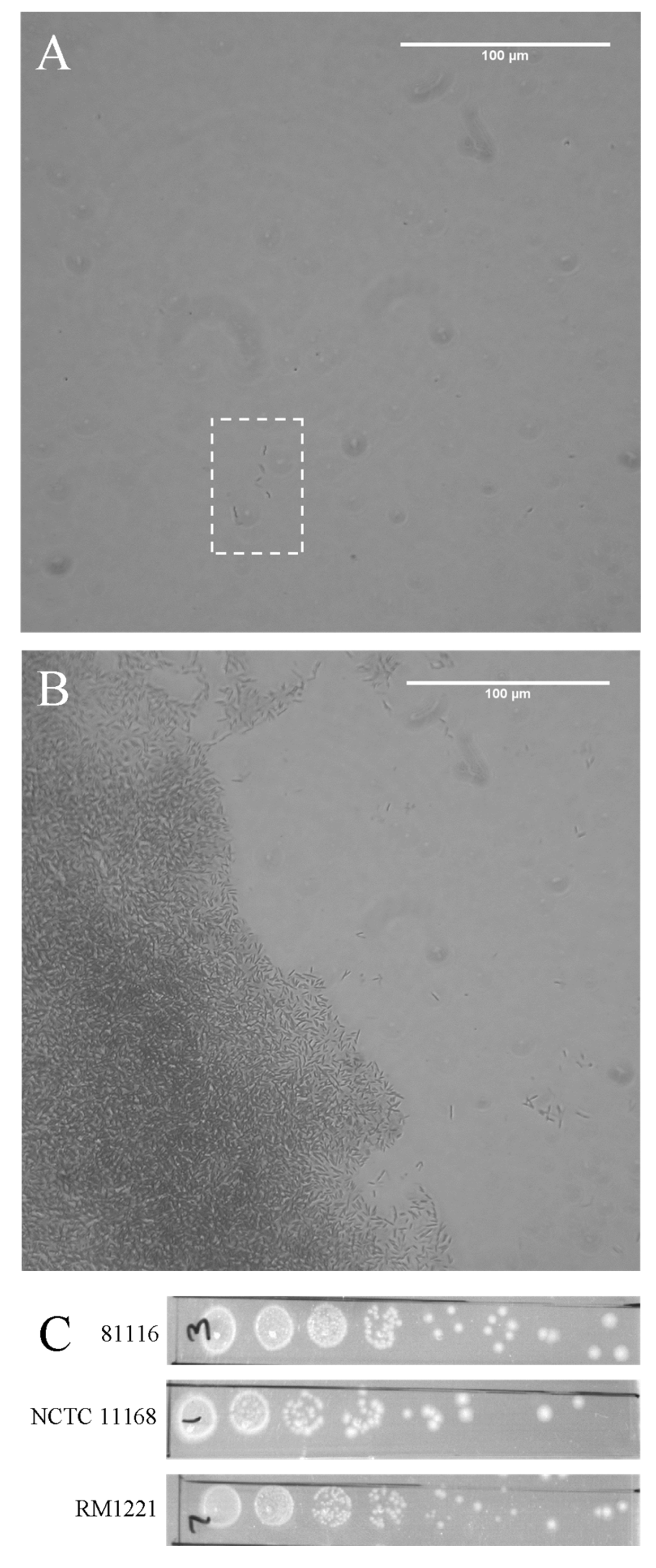

Supplement: S1 Fig — (A) and (B) show representative images of the air/liquid interface of a glass slide following 48 hours of static incubation at 37°C in aerobic conditions. (A) shows a slide incubated with RM1221 cells and (B) shows a slide incubated with NCTC 11168. The highlighted area in (A) shows potentially attached RM1221 cells, although no progression to microcolony formation is observed. (C) shows representative images of spot plates following 48 hour static incubation at 37°C in aerobic conditions. (TIF) [file pone.0121680.s001.tif]

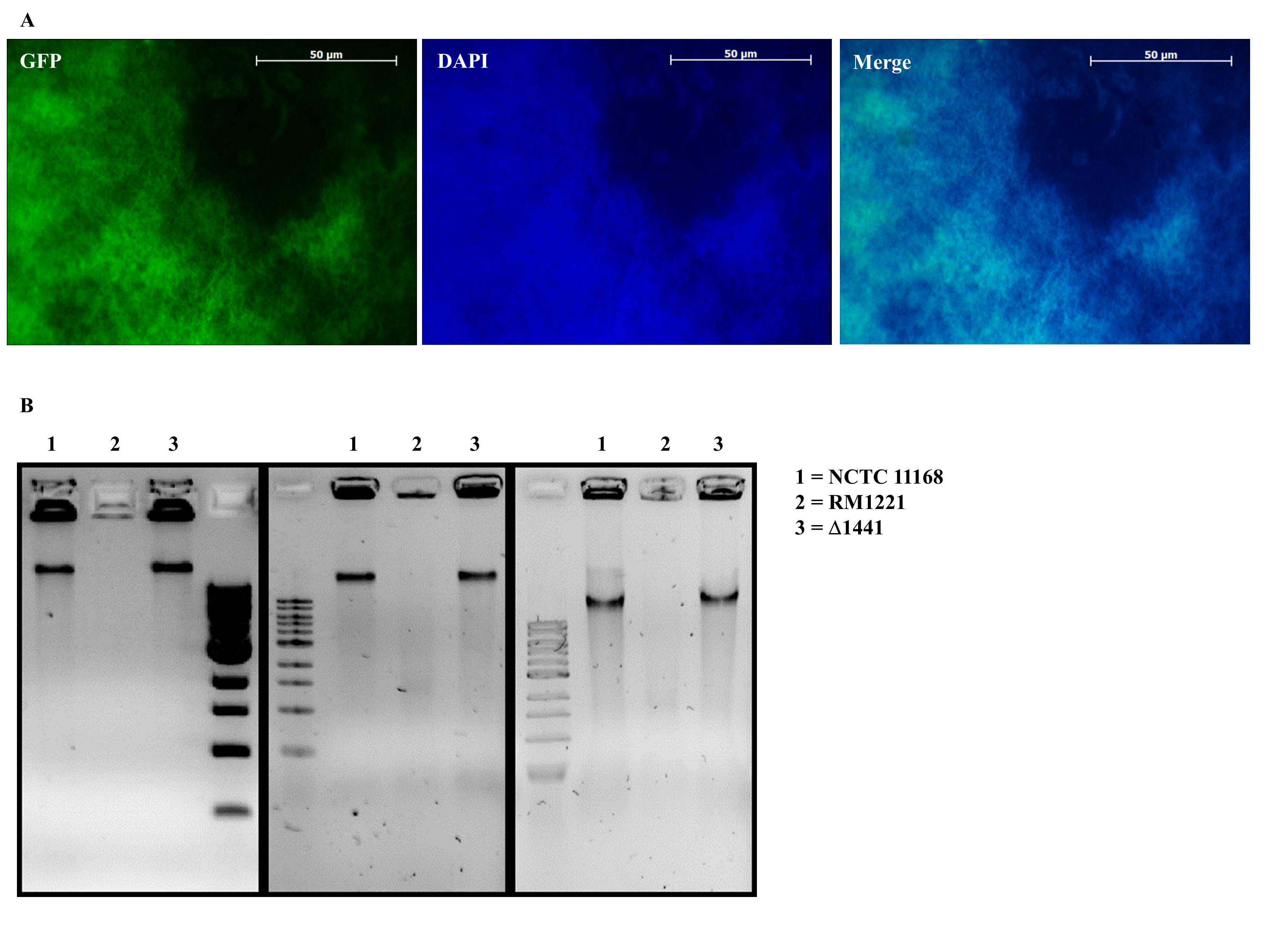

Supplement: S2 Fig — (A) Representative image of Green fluorescent protein (GFP)-expressing NCTC 11168 biofilms (strain NCTC 11168 GFP+, see Table 1) counter stained with DAPI. A diffuse blue dye can be seen around the GFP-expressing cells suggesting that there is a large quantity of eDNA present within the mature biofilm. (B) Three biological replicates showing ethidium bromide-stained DNA isolated from biofilm samples from strains NCTC 11168, RM1221, and Δ1441 after agarose gel electrophoresis. (TIF) [file pone.0121680.s002.tif]

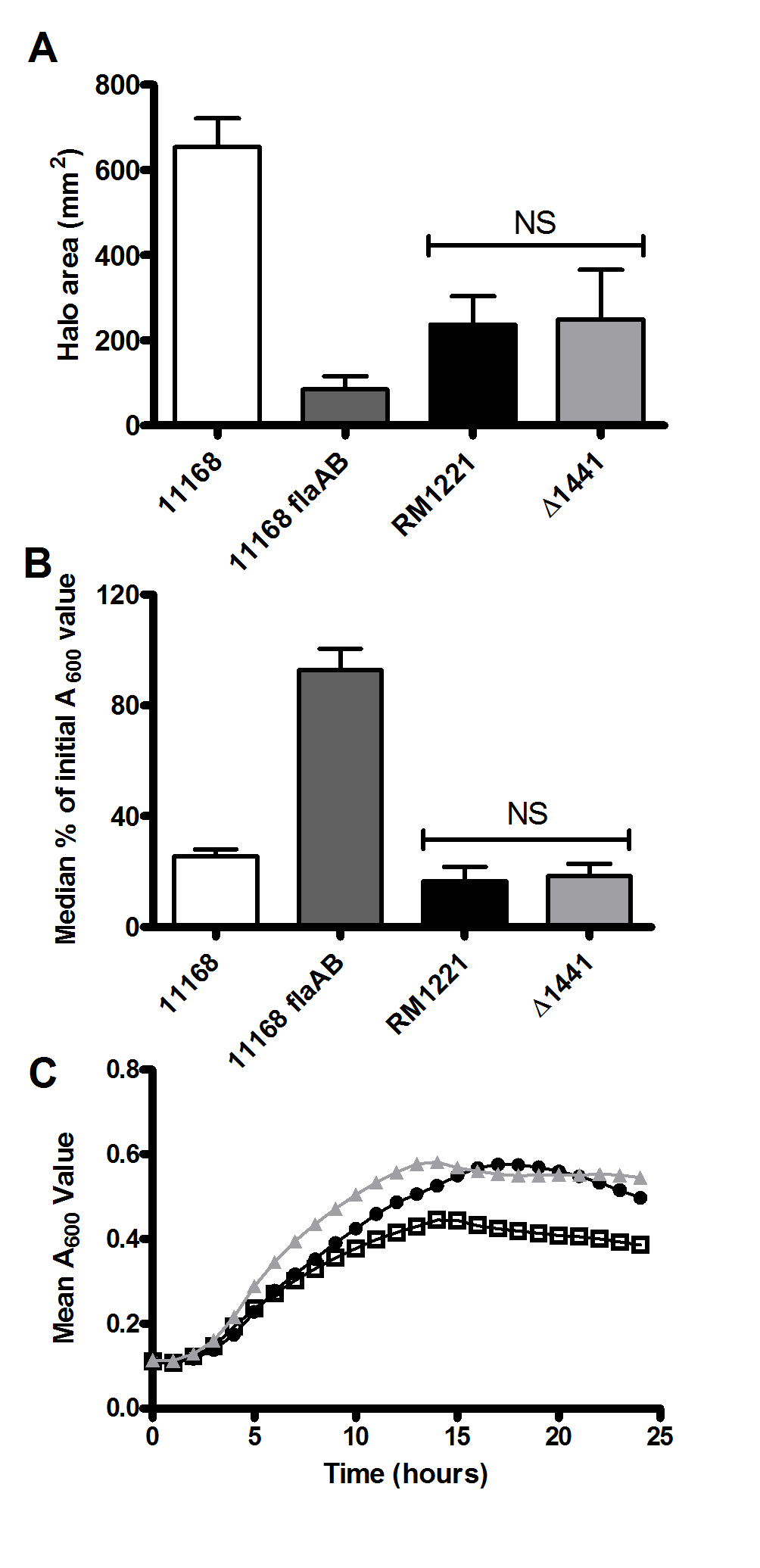

Supplement: S3 Fig — C. jejuni strains NCTC 11168 (white), its non-motile ΔflaAB mutant (dark grey), RM1221 (black bars) and the Δ1441 mutant (light grey) were compared for their ability to swarm (A) and autoagglutinate (B). In both tests no statistical difference was observed between Δ1441 and the wild-type. Panel C shows growth over a 24 hour period for Δ1441 (light grey triangles), RM1221 wild-type (black circles) and NCTC 11168 (white squares). Bars represent the median, error bars show range and significance was measured using Mann-Whitney tests. (TIF) [file pone.0121680.s003.tif]

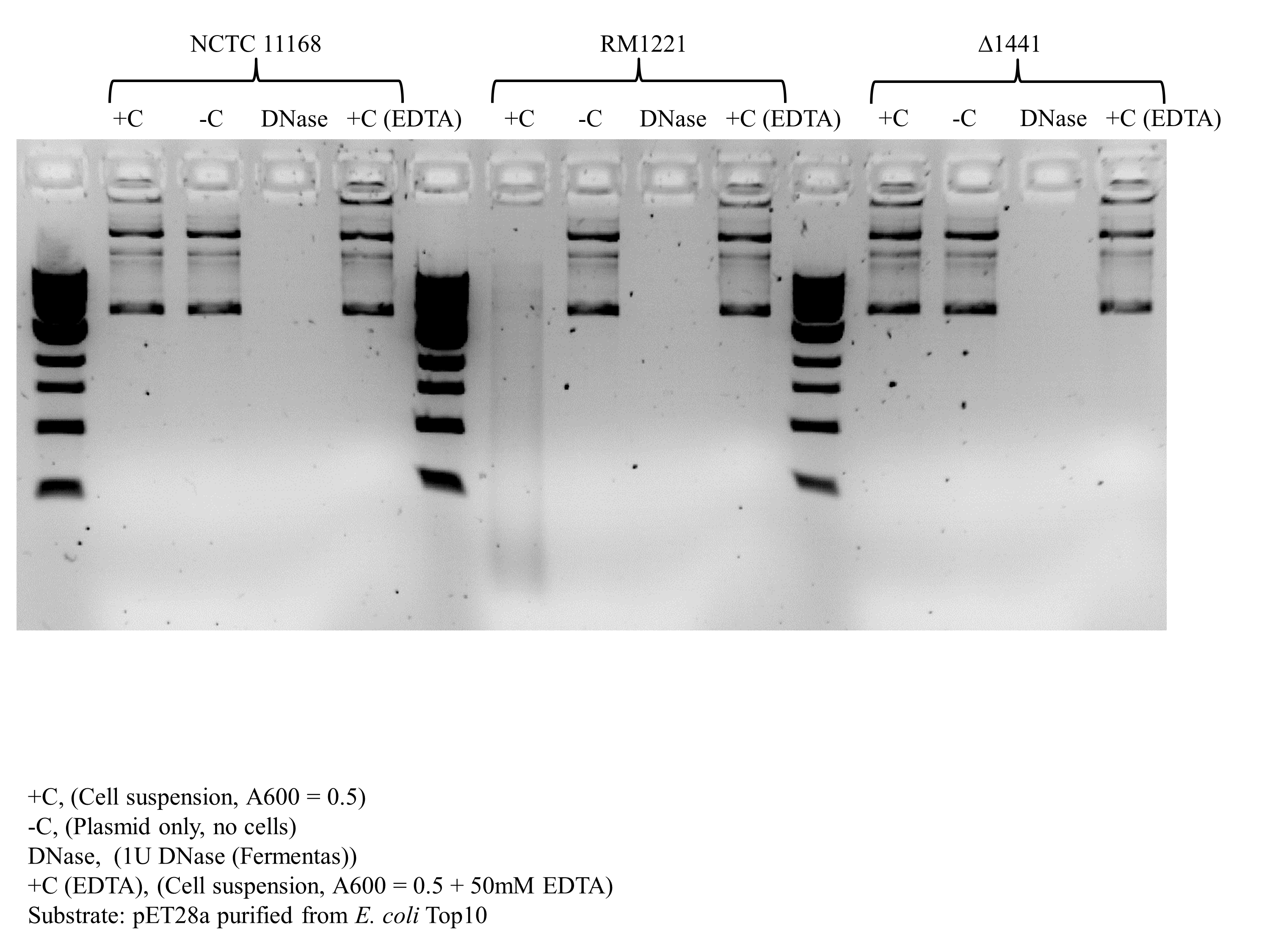

Supplement: S4 Fig — Plasmid DNA (70 ng) was incubated with cell suspensions for 60 minutes at 37°C prior to agarose gel electrophoresis. Plasmid DNA is almost entirely degraded in reactions containing RM1221 cell suspension, but not NCTC 11168 or Δ1441. (TIF) [file pone.0121680.s004.tif]
